# The renal pelvis urobiome in the unilateral kidney stone patients revealed by 2bRAD-M

**DOI:** 10.1186/s12967-022-03639-6

**Published:** 2022-09-24

**Authors:** Sen-Yuan Hong, Yuan-Yuan Yang, Jin-Zhou Xu, Qi-Dong Xia, Shao-Gang Wang, Yang Xun

**Affiliations:** Department of Urology, Tongji Hospital, Tongji Medical College, Huazhong University of Science and Technology, Wuhan, China

**Keywords:** Kidney stone disease, Renal pelvis urine, Urobiome, 2bRAD-M

## Abstract

**Background:**

The pathogenesis of kidney stone disease (KSD) is not fully understood, and potential contributing factors remain to be explored. Several studies have revealed that the urinary microbiome (urobiome) of stone formers was distinct from that of healthy individuals using 16S rRNA gene sequencing, most of which only provided microbial identification at the genus level. 2bRAD sequencing for Microbiome (2bRAD-M) is a novel sequencing technique that enables accurate characterization of the low-biomass microbiome at the species resolution. We aimed to apply 2bRAD-M to profile the renal pelvis urobiome of unilateral kidney stone patients and compared the urobiome with and without stone(s).

**Method:**

A total of 30 patients with unilateral stones were recruited, and their renal pelvis urine from both sides was collected. A ureteroscope was inserted into the renal pelvis with stone(s) and a ureteral catheter was placed into the ureteroscope to collect renal pelvis urine. This procedure was repeated again with new devices to collect the urine of the other side. 2bRAD-M was performed to characterize the renal pelvis urobiome of unilateral stone formers to explore whether microbial differences existed between the stone side and the non-stone side.

**Results:**

The microbial community composition of the stone side was similar to that of the non-stone side. Paired comparison showed that *Corynebacterium* was increased and *Prevotella* and *Lactobacillus* were decreased in the stone side. Four species (*Prevotella bivia, Lactobacillus iners, Corynebacterium aurimucosum, and Pseudomonas sp_286*) were overrepresented in the non-stone side. 24 differential taxa were also identified between two groups by linear discriminant analysis effect size (LEfSe). Extensive and close connections among genera and species were observed in the correlation analysis. Moreover, a random forest classifier was constructed using specific enriched species, which can distinguish the stone side from the non-stone side with an accuracy of 71.2%.

**Conclusion:**

This first 2bRAD-M microbiome survey gave an important hint towards the potential role of urinary dysbiosis in KSD and provided a better understanding of mechanism of stone formation.

**Supplementary Information:**

The online version contains supplementary material available at 10.1186/s12967-022-03639-6.

## Introduction

Kidney stone disease (KSD) is a common disease of urinary tract with increasing prevalence and a high risk of recurrence which poses a substantial healthcare burden [1, 2]. Although great advances have been achieved in the surgical techniques for stone removal, the development of effective medical drugs for treatment and prevention of KSD remains stagnant, since the detailed mechanisms of stone formation are still unclear. Calcium oxalate (CaOx) is the most dominant type of stones, followed by calcium phosphate (CaP), struvite, uric acid, and cystine [3]. Hypercalciuria and hyperoxaluria are major risk factors for stone formation, and supersaturation of the urine act as driving forces for crystallization [4]. However, idiopathic CaOx stone formers shared similarity in urine chemistries with healthy individuals [5]. Besides, most patients have unilateral stone disease, suggesting that supersaturation alone does not necessarily trigger stone formation. Hence, there must be other factors contributing to KSD.

With advanced techniques in sequencing and culturomics, the urinary tract has been discovered to harbor a large and diverse population of microbes [6–8]. The urinary microbiome (urobiome) was proven to be associated with several urologic diseases, such as urinary incontinence, overactive bladder, and urologic tumors [9, 10]. Thus, the role of the urobiome in KSD has attracted increasing research interest. Some researchers profiled the urobiome in stone formers by 16S rRNA gene sequencing and observed distinct microbial differences between stone formers and healthy individuals [11–14]. In addition, Dornbier et al. found several bacterial species were dominant in the paired bladder urine and stone homogenate from the same patient using 16S rRNA gene sequencing and enhanced quantitative urine culture (EQUC) [15]. These studies suggest the urobiome might be implicated in stone formation.

All current urobiome-related studies in KSD performed 16S rRNA gene sequencing to investigate the microbial community composition in the urine. As this sequencing technique only sequences 16S rRNA genes of bacteria, it generally provides taxonomic classification up to the genus level, which is difficult to achieve precise and reliable microbial identification at the species level. Although whole metagenome shotgun sequencing allows to sequence the entire genome from samples to resolve species-level taxonomy, it is much more costly and usually requires a great amount of DNA as starting material. However, the urobiome represents a relatively low biomass compared to the gut microbiome. We therefore sought to apply a method which can produce accurate and species-resolution taxonomic profiles to characterize the low-biomass urobiome in KSD. 2bRAD sequencing for Microbiome (2bRAD-M) is a novel sequencing approach to study the microbiome [16]. This method digests the genomic DNA of the samples using type IIB restriction enzymes to yield iso-length DNA fragments [17, 18]. The fragments were amplified for sequencing and mapped into species-specific 2bRAD markers for microbial characterization and quantification. 2bRAD-M has proven its ability to profile the low-biomass microbiome at the species level with high fidelity and low costs [16].

The present study enrolled patients with unilateral stones and collected renal pelvis urine from both sides. We performed 2bRAD-M to profile the renal pelvis urobiome and aimed to determine if the urobiome in the renal pelvis is different between kidneys with and without stone(s). To our best knowledge, it is the first attempt to explore the pelvis urobiome in unilateral stone formers at the species resolution using 2bRAD-M.

## Methods

### Recruitment of patients

In order to minimize the confounding factors that might affect the urobiome, strict exclusion criteria were established: urinary tract infections (UTIs), other urologic disease, history of major urological surgery, treatment with antibiotics within 4 weeks, urinary catheterization within 4 weeks. Ultimately, we recruited a total of 30 unilateral stone formers with an initial stone episode at Tongji Hospital. All patients were diagnosed by computed tomography and received percutaneous nephrolithotomy. The removed stones obtained during endoscopic surgery were sent for chemical composition analysis. The patients’ clinical information was also collected.

### Sample collection and processing

**Fig. 1:**
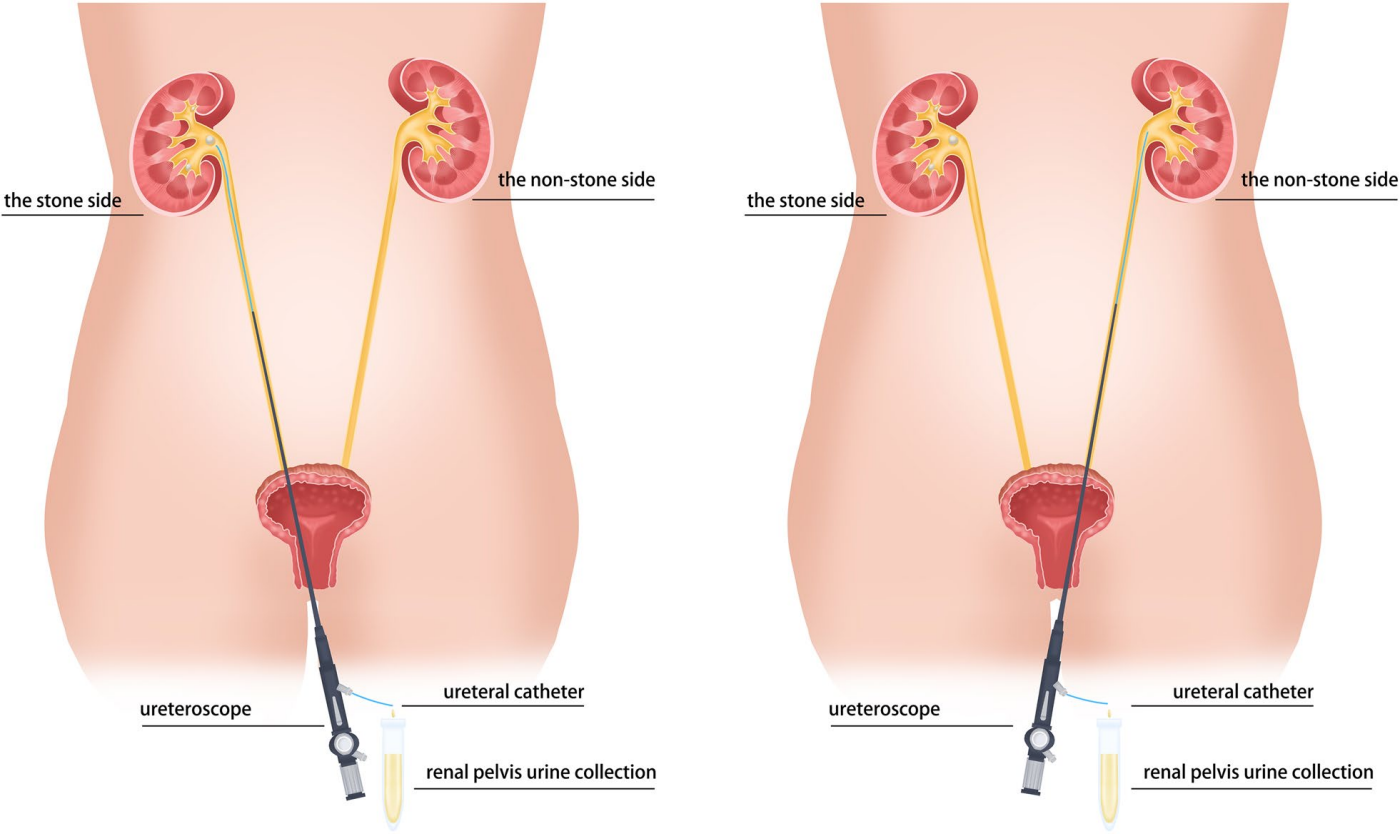
A graphical representation of renal pelvis urine collection. A ureteroscope was gently inserted into the renal pelvis of the stone side and a ureteral catheter was placed into the ureteroscope to collect renal pelvis urine. Then, a new ureteroscope and a new ureteral catheter were used and this procedure was repeated to collect the renal pelvis urine of the non-stone side in the same patients

Renal pelvis urine collection was approved by Ethical Review Board of Tongji Hospital, Tongji Medical College, Huazhong University of Science and Technology (2021S130). Informed consents were obtained from patients for the use of their samples. To prevent the mixture of bladder urine, the bladder was voided by urethral catheter before renal pelvis urine collection. Next, a ureteroscope was gently inserted into the renal pelvis with stone(s) and a ureteral catheter was placed into the ureteroscope to collect renal pelvis urine. After 5 ml of urine collection was completed, the ureteroscope and ureteral catheter were withdrawn. Subsequently, a new ureteroscope and a new ureteral catheter were used and this procedure was repeated to collect the urine of the other renal pelvis without stone(s) in the same patients. The workflow for collecting renal pelvis urine samples is shown in Fig. 1. The urine samples were stored under −80 °C within 1 h from collection. The whole process was performed under aseptic conditions.

### DNA extraction, library preparation and sequencing

TIANamp Micro DNA Kit (Tiangen) was used to extract the genomic DNA. 2bRAD libraries were prepared according to previous studies [16, 19]. First, the genomic DNA was digested with 4 U of BcgI restriction enzyme (NEB) at 37 °C for 3 h. Next, a reaction volume of 20 ul containing 10 μl of digested product, 0.2 μM each of library-specific adaptors (Ada1 and Ada2), 1 mM ATP (NEB), 1 × T4 DNA Ligase Buffer, and 800 U T4 DNA ligase (NEB) was used for ligation reaction at 4 °C for 16 h. Then, heat inactivation of BcgI was performed at 65 °C for 20 min. After that, ligation products were submitted to PCR amplification in a 40 ul reaction volume consisted of 7 μl ligated DNA, 0.1 μM each primer (Primer1 and Primer2 for Illumina), 0.3 mM dNTP, 1 × Phusion HF buffer, and 0.4 U Phusion high-fidelity DNA polymerase (NEB). Each PCR reaction was carried out for 16–28 cycles as follows: 98 °C for 5 s, 60 °C for 20 s, and 72 °C for 10 s and then a final extension of 10 min at 72 °C. The library products were purified with the QIAquick PCR purification kit (Qiagen) and subjected to sequencing via Illumina HiSeq X™ Ten platform. Library construction and Illumina sequencing were performed at OE BioTech Co., Ltd., Qingdao. All adaptor and primer sequences are provided at Additional file 1: Table S1.

### Sequencing processing and quantitative analysis

Raw reads were filtered to extract the digested fragment called “enzyme reads” according to BcgI restriction enzyme recognition site. Clean reads were obtained from enzyme reads with the following criteria: (1) removing reads with greater than 8% unknown bases; (2) removing reads containing more than 20% of low-quality bases (Q-value ≤ 20). The 2bRAD-M computational pipeline (https://github.com/shihuang047/2bRAD-M) was used to perform the taxonomic profiling, whose foundation is the unique 2bRAD tag database (2b-Tag-DB) which contains taxa-specific BcgI-derived tags identified from 173,165 microbial genomes (including bacteria, fungi, and archaea). First, clean reads were mapped against the prebuilt 2b-Tag-DB to identify microbial taxa presented in a sample. The G score was derived for each species identified within a sample to control the false-positive discovery with the following formula: G score _species i_ = $\sqrt{{S}_{i}\times {t}_{i}}$ (S: the number of reads assigned to all 2bRAD tags of species i within a sample; t: the number of all 2bRAD tags of species i that have been sequenced within a sample). The G score was proven to be more sensitive as the threshold than the relative abundance estimated by Bracken [16], and the threshold of G score for a false-positive discovery of species was set as 10. Thus, candidate taxa were screened with a minimum G score threshold of 10. For accurate quantitative estimates of identified taxa, a secondary 2b-Tag-DB was constructed only based on those candidate taxa, which contains more specific 2bRAD tags for each candidate taxa than those in the default 2b-Tag-DB. All the reads were then remapped against this sample-specific 2b-Tag-DB to evaluate the relative abundance of candidate taxa. Subsequently, the relative abundance of a given species was calculated with the following formula: Relative abundance _species i_ = $\frac{{S_{{\text{i}}} /T_{{\text{i}}} }}{{\sum\limits_{{{\text{i}} = 1}}^{{\text{n}}} {S_{{\text{i}}} /T_{{\text{i}}} } }}$(S: the number of reads assigned to all 2bRAD tags of species i within a sample; T: the total number of 2bRAD tags of species i). Finally, a taxonomic abundance profile was obtained.

### Microbial diversity analysis and identification of differential taxa

Based on the taxonomic abundance profiles, alpha diversity indices, such as Chao1, Shannon index, and Simpson index were calculated using the “vegan” package and visualized as boxplots [20]. Beta diversity was estimated by computing Bray–Curtis distance, Binary Jaccard distance, and Euclidean distance algorithms using the “vegan” package and visualized as principal coordinate analysis (PCoA). A Venn diagram was used to display the unique and common species between the stone side and the non-stone side. Linear discriminant analysis (LDA) effect size (LEfSe) was performed to identify taxa differentially represented between the stone side and the non-stone side, and the threshold on the logarithmic LDA score for discriminative features was 2.0 [21]. To discriminate the stone side from the non-stone side, a random forest model using tenfold cross-validation with 10 repeats was generated using the “randomForest” package, and the cross-validation error curve was obtained [22]. The point with the minimum cross-validation error was set as the cut-off point, and the minimum error plus the standard deviation (SD) at that point was used as the cut-off value. We listed all sets of biomarkers with the error less than the cut-off value and define the optimal set as the set with the smallest number of species. To evaluate the discriminatory ability of the random forest model, the receiver operating characteristic curve (ROC) was constructed and the area under the ROC curve (AUC) was calculated using the “pROC” package [23]. The probability of disease (POD) index refers to the ratio between the number of randomly generated decision trees that predicted a sample as the stone side and that of the non-stone side [24]. The optimal set of species was finally used for the calculation of POD index for the cohort.

### Statistical analysis

Statistical analyses were performed with the SPSS (version 26) and R software (version 4.1.1). Among clinical parameters, categorical variables were expressed as percentages, and continuous variables as mean ± SD. For group comparisons of alpha diversity and microbial communities, the paired Wilcoxon test was used. For beta diversity, statistical comparisons of Bray–Curtis distance, Binary Jaccard distance, and Euclidean distance were conducted by permutational multivariate analysis of variance (PERMANOVA) to evaluate differences between the two groups. In addition, the correlation between different species was calculated with the Spearman correlation analysis based on the relative abundance. P-value < 0.05 was considered as statistically significant.

## Results

### Clinical characteristics of the enrolled patients

30 patients with unilateral kidney stones were recruited for the study. Demographic and clinical characteristics of the enrolled patients were listed in Table 1. The patients comprised 21 men and 9 women, with an average age of 49.3. Calculi were found on the left side in 19 patients, and on the right side in 11 patients. A single stone was found in 16 and multiple stones were found in 14 of 30 cases. Stone composition was available 18 patients (60%), among which 15 cases had calcium-based stones (CaOx or CaP) and only 3 cases have uric acid stones. In addition, approximately 27% of patients presented with comorbidities, and most of them have hypertension.

**Table 1:** Demographic and clinical characteristics of the enrolled patients (n = 30)

Parameter	Number (%) or mean ± SD
Age	49.3 ± 15.6
Gender	
Male	21 (70)
Female	9 (30)
Body mass index (kg/m^2^)	23.9 ± 2.6
Stone side	
Left	19 (63.3)
Right	11 (36.7)
Stone count	
Single	16 (53.3)
Multiple	14 (46.7)
Stone composition	
CaOx	8 (26.7)
CaOx + CaP	7 (23.3)
Uric acid	3 (10)
NA	12 (40)
Comorbidities	
Hypertension	7 (23.3)
Diabetes	1 (3.3)

### Biodiversity of the pelvis urobiome

**Fig. 2:**
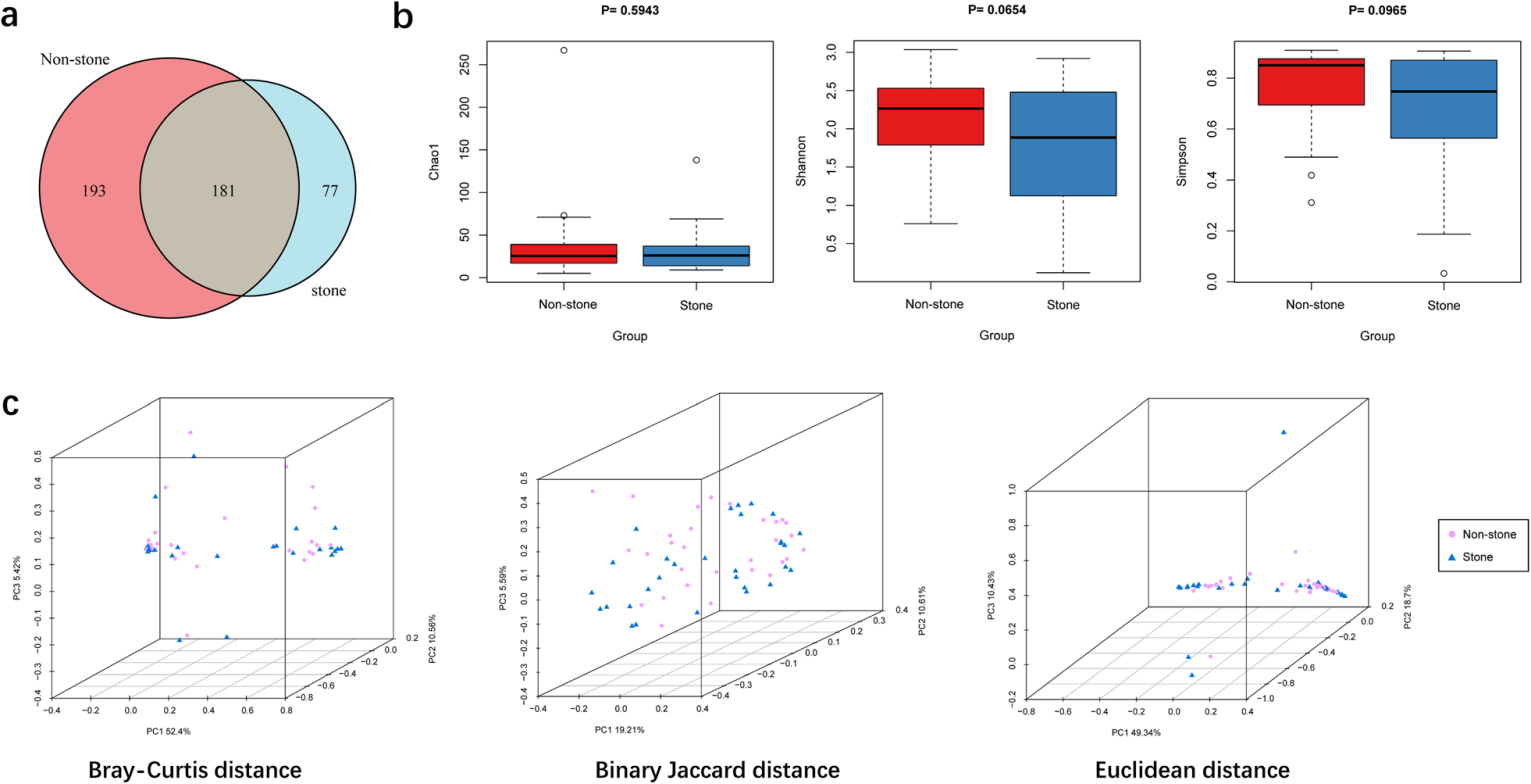
Microbial diversity and structure of the renal pelvis urobiome between the stone group and the control group. **a** A Venn diagram exhibited the shared and unique species between the two groups. **b** Comparison of alpha diversity (Chao1, Shannon index, and Simpson index) between the two groups. **c** Comparison of beta diversity between the two groups based on Bray–Curtis distance, Binary Jaccard distance and Euclidean distance. 3D-PCoA plot showed the clustering of bacterial taxa in the two groups based on three distance matrices, with each point corresponding to a sample and colored according to the sample type. PERMANOVA revealed that the urobiome of the stone side was similar to that of the non-stone side

The information of quality control during sequencing is showed in Additional file 2: Table S2. A total of 430 million clean reads were obtained from 60 renal pelvis urine samples with an average of 7.16 million reads per sample. The clean reads were classified into 451 unique bacterial species based on alignment against 2b-Tag-DB. As shown in the Venn diagram, the stone side and the non-stone side shared 181 (40.13%) species in common (Fig. 2a). 77 (17.07%) unique species were identified in the stone side, and 193 (42.79%) in the non-stone side. For alpha diversity, Chao1 was calculated to assess the community richness of the renal pelvis urobiome and the Shannon index and Simpson index was calculated to evaluate community diversity. Chao1, Shannon index and Simpson index showed no significant difference between two groups (Fig. 2b). Although the differences were non-significant, the non-stone side tended to have higher bacterial diversity than the stone side. For beta diversity, PCoA revealed no obvious differences in bacterial composition between two groups based on Bray–Curtis distance (p = 0.604), Binary Jaccard distance (p = 0.694), and Euclidean distance (p = 0.664) (Fig. 2c). The renal pelvis urine samples were also divided into four groups: the stone side of male patients (SM), the stone side of female patients (SF), the non-stone side of male patients (NSM), and the non-stone side of female patients (NSF). Through diversity analysis, there were no differences between SM and SF, or between SM and NSM, or between SF and NSF (Additional file 3: Fig. S1).

### Bacterial community composition in the pelvis urobiome

**Fig. 3:**
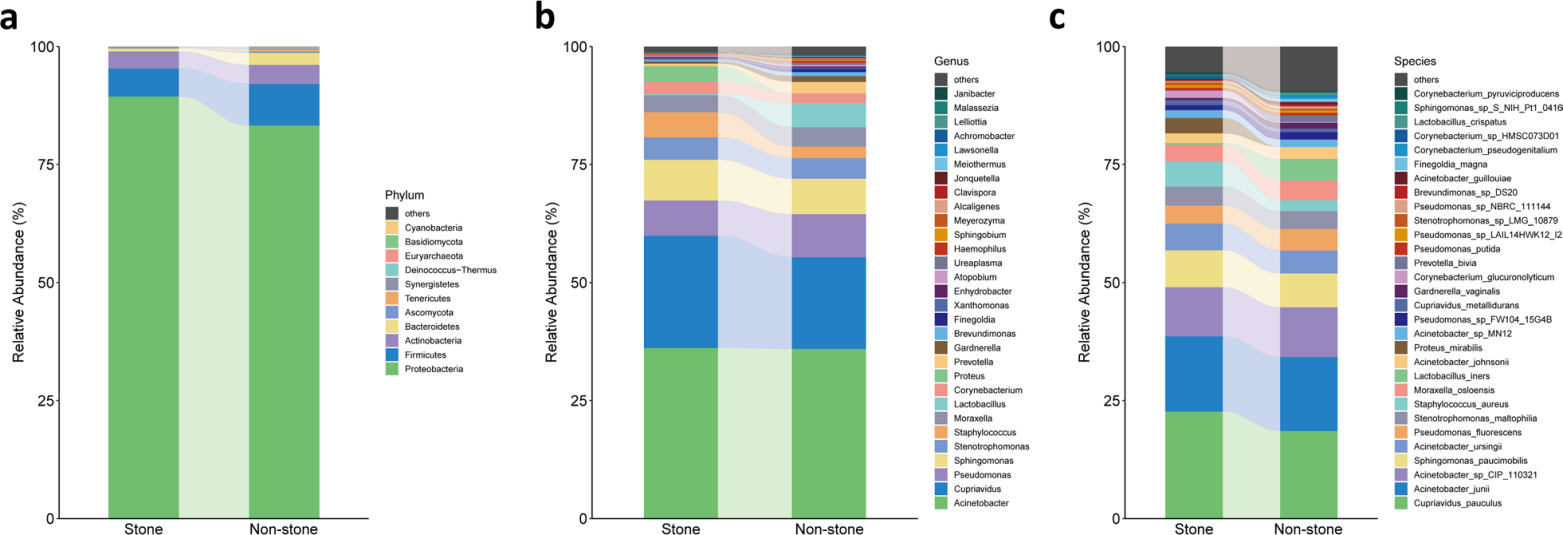
Bacterial abundance and distribution in the two groups. The relative abundance of the bacterial phyla (**a**), the top 30 most abundant genera (**b**), and species (**c**) is represented in the barplot

10 and 12 phyla were identified in the stone and non-stone sides, respectively. Proteobacteria was the dominant phylum for both the groups (89.44 and 83.28%), followed by Firmicutes (5.90 and 8.79%) and Actinobacteria (3.65 and 4.07%) (Fig. 3a). 102 and 134 genera were detected in stone and non-stone sides, respectively. The dominant bacterial genus in the stone side was *Acinetobacter* (36.15%), followed by *Cupriavidus* (23.77%), *Sphingomonas* (8.63%), *Pseudomonas* (7.45%) and *Staphylococcus* (5.37%). In the non-stone side, the major genera were *Acinetobacter* (35.98%), *Cupriavidus* (19.34%), *Pseudomonas* (9.15%), *Sphingomonas* (7.51%) and *Lactobacillus* (5.18%) (Fig. 3b). At the species level, *Cupriavidus pauculus*, *Acinetobacter junii*, *Acinetobacter sp_CIP_110321*, *Sphingomonas paucimobilis*, *Acinetobacter ursingii* were the top 5 most abundant taxa in both groups (Fig. 3c).

### Differential abundances of bacterial taxa between the stone side and the non-stone side

**Fig. 4:**
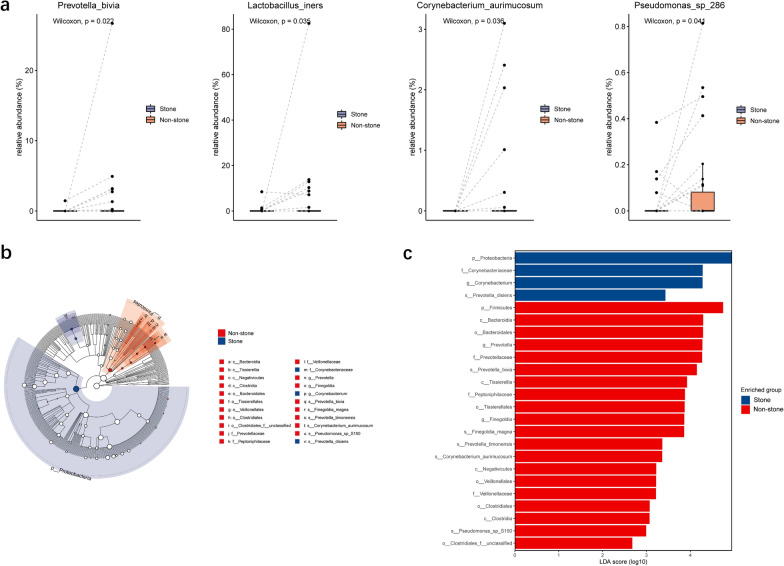
Differential abundances of bacterial taxa between the two groups. **a** The boxplot showed four species were increased in the non-stone side through paired Wilcoxon test. **b** The Cladogram represents the taxonomic hierarchical structure of the identified 24 discriminative biomarkers using LEfSe. **c** The histogram of LDA score showed 24 biomarkers with significant differences between the two groups. LDA score represented the influencing degree of biomarkers

To identify the differentially represented taxa in the stone side and the non-stone side, we compared the relative abundance of taxa between two group using the paired Wilcoxon test (Table 2). At the phylum level, the stone side exhibited increased abundance of Proteobacteria and decreased abundance of Bacteroidetes, Actinobacteria and Firmicutes compared to the non-stone side. At the genus level, a significantly higher abundance of *Corynebacterium* and lower abundance of *Prevotella* and *Lactobacillus* were observed in the stone side. At the species level, we observed that *Prevotella bivia*, *Lactobacillus iners*, *Corynebacterium aurimucosum*, and *Pseudomonas sp_286* were enriched in the non-stone side compared to the stone side (Fig. 4a).

**Table 2:** Comparison of average relative abundance of pelvis urinary microbiome between the stone side and the non-stone side at the genus and species levels

Taxa		Average relative abundance (%)		
		Stone	Non-stone	P-value
Phylum	Bacteroidetes	0.636	2.543	0.013
	Proteobacteria	89.440	83.277	0.028
	Actinobacteria	3.651	4.067	0.033
	Firmicutes	5.895	8.793	0.033
Genus	*Prevotella*	0.565	2.435	0.010
	*Lactobacillus*	0.345	5.181	0.021
	*Corynebacterium*	2.535	1.995	0.033
Species	*Prevotella bivia*	0.049	1.406	0.022
	*Lactobacillus iners*	0.343	4.565	0.035
	*Corynebacterium aurimucosum*	0	0.297	0.036
	*Pseudomonas sp_286*	0.026	0.094	0.041

We also applied LEfSe to determine the differentially abundant taxa in the two groups. LEfSe identified 24 discriminative features (LDA score ≥ 2.0) with significant different relative abundance between the stone side and the non-stone side (Fig. 4b, c). At the genus level, urobiome of the stone side was enriched with *Corynebacterium*, while urobiome of the non-stone side was enriched with *Prevotella* and *Finegoldia*. The taxa at the species level that differentiated the two groups were *Prevotella disiens* in the stone side and *P. bivia*, *Finegoldia magna*, *Prevotella timonensis*, *C. aurimucosum*, and *Pseudomonas sp_S150* in the non-stone side.

### Connections among the top abundant genera and species

**Fig. 5:**
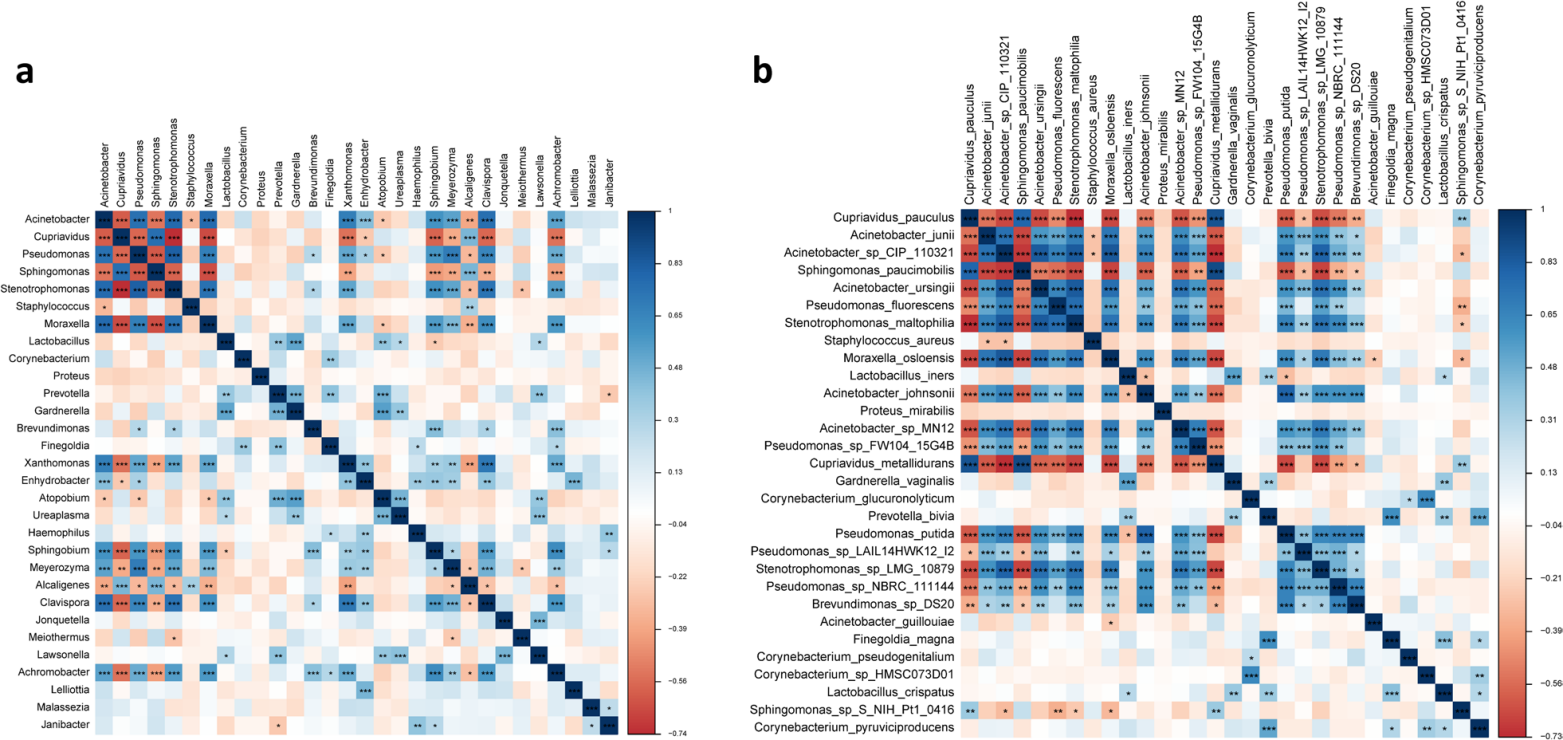
Heatmap of Spearman correlation analysis of the top 30 most abundant genera (**a**) and species (**b**)

To assess the association among the top 30 most abundant genera and species, the Spearman correlation analysis was performed. Significant positive correlations and negative correlations were observed among different taxa (Fig. 5a, b). For example, *Acinetobacter*, the most dominant genus, was positively correlated with several opportunistic pathogens, such as *Stenotrophomonas* (R = 0.78, P = 3.50 × 10^–13^) and *Pseudomonas* (R = 0.75, P = 6.38 × 10^–12^), and was negatively correlated with *Cupriavidus* (R = −0.61, P = 2.40 × 10^–7^) and *Sphingomonas* (R = −0.53, P = 1.13 × 10^–5^). The beneficial genera *Lactobacillus* was positively correlated with *Prevotella* (R = 0.37, P = 3.34 × 10^–3^).

### Classification of stone status using bacterial species‑level biomarkers

**Fig. 6:**
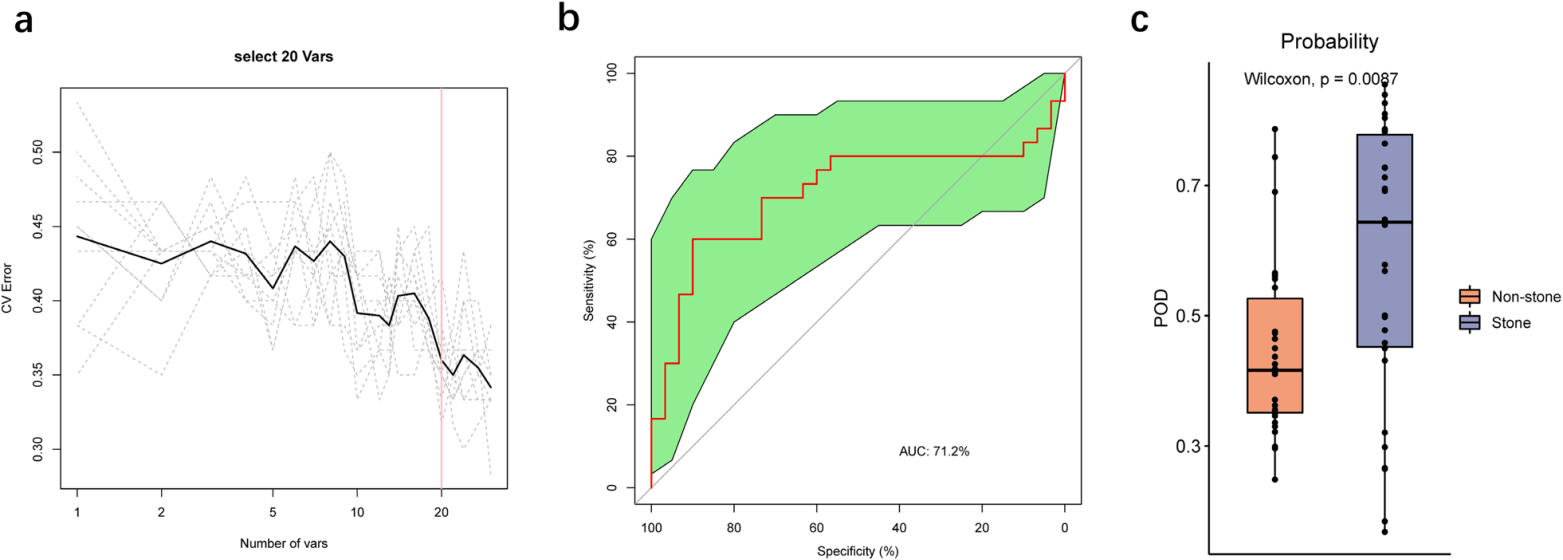
Identification of species-based markers of the stone group by a random forest model. **a** The cross-validation error curve showed that 30 species markers were selected as the optimal marker set by tenfold cross-validation. **b** The average AUC value reached 71.2% between the two groups. **c** The POD value was significantly increased in the stone group versus the control group

We constructed a random forest classifier that could specifically identify the stone side samples from the non-stone side samples. We selected the top 30 most abundant species to construct the classification models. The cross-validation error curve was obtained from 10 trials of tenfold cross-validation. 20 species markers were selected as the optimal marker set to distinguish the stone side from the non-stone side (Fig. 6a and Additional file 4: Table S3). ROC analysis was performed to assess the performance of these optimal marker models, and the average AUC value achieved 71.2% between the stone side and the non-stone side (Fig. 6b). The POD value was significantly increased in the stone side compared to the non-stone side (p = 0.0087), suggesting that the POD based on microbial species markers achieved a powerful diagnostic potential for the stone side from the non-stone side (Fig. 6c).

## Discussion

KSD is a complex disorder with a multifactorial etiology involving environmental and genetic factors. The application of 16S rRNA gene sequencing and the EQUC has revealed that a complex and diverse community of microbes inhabiting the urinary tract of healthy individuals [6–8]. Since then, the urobiome has been verified to have links with some urologic diseases [9, 10]. Increasing attention has been directed towards the role of the urobiome in KSD. It is well recognized that struvite stones, namely infective stones, were caused by urea-producing bacteria, such as *Proteus mirabilis* [25]. However, clinical studies found that the majority of bacteria isolated from all types of stones were non-urea-producing bacteria, and the most common bacterium isolated from urine and stone samples of patients was *Escherichia coli*, suggesting that metabolic stones (CaOx, CaP, uric acid, etc.) were also related to urinary bacteria [26, 27]. Recently, a distinct difference in microbial community composition was observed between stone formers and healthy individuals, further hinting at the potential importance of the urobiome in stone formation [11–14]. But all these studies applied 16S rRNA gene sequencing to profile the urobiome, and the results mostly do not resolve taxa below the genus level. In addition, most studies conducted comparative analysis of the bladder urobiome between stone formers and healthy controls. However, renal pelvis urine is more reflective of the microbiota colonizing the kidney than bladder urine in terms of the anatomic location. Some researchers have collected renal pelvis urine of stone formers for sequencing [11, 13, 28], but there are currently no studies with large sample sizes that compare the renal pelvis urobiome with and without stone(s) in unilateral stone formers. Herein, we aim to characterize the renal pelvis urobiome of unilateral stone formers to explore whether microbial differences existed between the stone side and the non-stone side using 2bRAD-M, a novel sequencing technique that can depict the landscape of microbial community at the species level with high accuracy and low costs.

Both alpha diversity and beta diversity indicated that the overall microbial composition of the renal pelvis urine with stone(s) was similar to that without stone(s). There may be several possibilities for these results. First, the contamination of bladder during urine collection could lead to a high degree of similarity between the stone side and the non-stone side. Second, although we recruited unilateral stone formers with an initial stone episode, we cannot exclude the possibility that the patients might have bilateral stone formation before and the small stones may be eliminated in the urine. Since the non-stone side might develop stone formation before, it is easy to understand that both sides shared similarity with each other. Third, the resemblance between the urobiome in the stone side and the non-stone side was consistent with the previous findings [13]. Liu et al. collected four types of urine samples in kidney stone patients, including bladder urine aspirated before bladder disinfection, newly formed bladder urine after bladder disinfection, kidney pelvis urine in the stone side after bladder disinfection, and kidney pelvis urine in the non-stone side after bladder disinfection. They have disinfected the bladder carefully to prevent contamination, but they found that four types of urine samples had similar microbial richness and diversity. Thus, we assumed that the diversity of the stone side was similar to that of the non-stone side in nature as they both came from the same subject. There might be some differential taxa between two groups, but the overall microbial communities in both groups did not differ significantly. Although Yang et al. found that the overall microbial composition of the stone side and the non-stone side was different, their study was limited by a very small sample size of just 4 patients [28].

The abundance profiles showed that the dominant bacterial composition at different taxonomic levels was quite similar between the two groups. Some bacterial genera, including *Acinetobacter*, *Pseudomonas*, *Sphingomonas*, and *Staphylococcus*, were also previously reported as dominant genera in renal pelvis urine of stone formers [13, 28]. Of note, *Acinetobacter*, *Pseudomonas*, and *Staphylococcus* are important opportunistic pathogen that can cause UTIs. Some studies revealed that *Acinetobacter* is overrepresented in the bladder urobiome of stone formers compared to controls [11, 12]. *Pseudomonas* and *Staphylococcus* were found to be dominant in stone homogenate, and *Pseudomonas aeruginosa* and *Staphylococcus epidermidis* were isolated from these samples via EQUC [15, 29]. Although the major genera *Cupriavidus* has not been reported in any previous study about the urobiome in KSD, it has been associated with other urologic diseases. For example, *Cupriavidus* was more abundant in the bladder cancerous tissues than paracancerous tissues [30]. The present study identified 451 bacterial species, and the top 5 most abundant species were *C. pauculus*, *A. junii*, *A. sp_CIP_110321*, *S. paucimobilis*, and *A. ursingii*. These species are mainly present mainly in the natural environment that have rarely been reported in human infections.

Through paired comparisons and LEfSe, we found that the genus *Corynebacterium* and the species *P. disiens* was increased and the genera *Prevotella* and *Lactobacillus* and the species *P. bivia*, *P. timonensis*, *L. iners*, *C. aurimucosum*, *F. magna*, *P. sp_286* and *P. sp_S150* were decreased in renal pelvis urobiome of the stone side versus the non-stone side. An increased level of *Corynebacterium* in renal pelvis urine with stone(s) was also previously observed [13]. Besides, *Corynebacterium* spp. were cultured and isolated from stone homogenate [15, 31]. *Corynebacterium urealyticum*, named for its potent ability to split urea, is confirmed to be related to urinary calculi [32, 33]. *Corynebacterium matruchotii*, an inhabitant in the oral cavity, is associated with dental calculi formation and induce calcium precipitation and apatite deposition in vitro [34]. It is well accepted that most CaOx stones form on a base of interstitial apatite deposits called Randall’s plaque [35]. These clues have pointed to the potential causative role of *Corynebacterium* in KSD. Notably, though, *C. aurimucosum* was enriched in the non-stone side.

*Lactobacillus* and *Prevotella* have been previously reported to be underrepresented in the bladder urobiome of stone fomers [11, 14]. *Lactobacillus* is the common resident of vagina that can provide a healthy vagina environment and prevent the colonization of pathogens through production of lactic acid and antimicrobial compounds [36]. *Lactobacillus* is also the dominant member of healthy bladder urobiome, and it could likewise inhibit the growth of urinary pathogens [8, 37]. Probiotics belonging to *Lactobacillus* spp. have been applied to prevent UTIs through intravaginal treatment, and a robust vaginal colonization with *Lactobacillus* with reduced recurrence rate was observed [38]. *L. iners* is a common *Lactobacillus* spp. in the vaginal microbiome and bladder urobiome, which was found to share 99.99% similarity between the vagina and bladder [39]. Due to its good adaptability, *L. iners* can maintain a relatively constant abundance under fluctuating environmental conditions, while other *Lactobacillus* spp. may not survive [40, 41]. Besides, *L. iners* prevents pathogens from obtaining important nutrients and inhibit their growth through activation of the innate immune system in vaginal epithelial cells [42]. Studies also showed that UTI risk is associated with depletion of *L. iners* [43]. The above evidences suggest *L. iners* might play a protective role in KSD. *Prevotella* is highly present across different body sites, including vaginal, gastrointestinal and urinary tract. Some *Prevotella* spp. are proficient producers of the short-chain fatty acids (SCFAs) [44]. SCFAs are major metabolites of bacterial fermentation that maintain the intestinal barrier integrity and exert anti-inflammatory effects, and it can regulate oxidative stress to prevent acute and chronic kidney injury through the gut-kidney axis [45, 46]. Since oxidative stress is closely involved in stone formation, it is no surprise that the intestinal microbiome of stone formers showed decreased levels of *Prevotella* [47, 48]. *P. bivia*, *P. timonensis*, and *P. disiens* are highly abundant species in vagina, all of which are associated with vaginal dysbiosis [49]. However, their role in urinary disease has not been reported and requires further exploration.

Correlation analysis has revealed extensive and close connections among different genera and species. The positively correlations indicate that some bacteria might support each other’s growth and colonization, while the negative correlations suggest that some bacteria might compete with other residents in the same niche to hinder their growth. In addition, 20 species from top 30 most abundant species were selected as the optimal marker set to construct a random forest model, which distinguished the stone side from the non-stone side with accuracies of 71.2%. The POD based on the 20 species markers also achieved a powerful classification potential. Since the species markers could be a potentially effective tool for predicting the stone group, the communities of these species might be implicated in stone formation.

Based on existing research on urobiome, it is unlikely that a single pathogen contributes to KSD. The renal pelvis might harbor diverse communities of microbes that are constantly in interaction with each other. Pathogenic bacteria might change urine chemistries to create a lithogenic environment, promote CaOx crystals growth and aggregation through surface structures, and induce the expression of pro-inflammatory proteins and stone matrix proteins in renal tubular epithelium to exacerbate the progress of stone formation [29, 50, 51]. Protective bacteria might have anti-inflammatory functions, and inhibit pathogenic bacteria colonization to maintain a balanced microbiome composition, favoring an anti-lithogenic environment. We assumed that a loss of protective bacteria and a consequent increase of pathogenic bacteria in the kidney, referred to as urinary dysbiosis, might be a key step in the process of KSD.

Our study had several limitations. First, sample size was modest, and 40% of samples lacked data on stone composition, which limited generalizability, comparison and subgroup analysis. A larger study population is needed to expand urobiome knowledge across stone types. Second, 2bRAD-M was unable to predict the potential functional pathways of the urobiome. The application of metagenomics will allow an insight into structural and functional information of the microbial communities. Finally, like most urobiome studies, our study was just descriptive in nature, failing to directly explore the functionality of the urobiome. Thus, it is difficult to determine the causal relationship between the urobiome and KSD. Longitudinal follow-up studies and experimental studies are necessary to whether the bacteria are contributors, bystanders, or consequences of stone formation.

## Conclusion

In summary, our study is the first one to apply 2bRAD-M, a novel microbial sequencing technology that can provide accurate taxonomic profiles to the species level for low-biomass samples, to characterize the urobiome of renal pelvis urine in unilateral stone formers. Overall, the renal pelvis urobiome with and without stone(s) shared similar microbial composition. Increased levels of *Corynebacterium* and reduced levels of *Prevotella* and *Lactobacillus* were observed in the stone side, and *P. bivia*, *L. iners*, *C. aurimucosum*, and *P. sp_286* were enriched in the non-stone side. The connections among genera and species were widespread in the urobiome, and some species can be used to discriminate the stone side from the non-stone side with high accuracy. We speculated that the urinary dysbiosis might play an important role in stone formation. However, further study is needed to gain deeper insight into the association between KSD and urobiome.

## Supplementary Information

**Additional file 1: Table S1.** The sequences of adaptors and primers used in 2bRAD-M (5’-3’)

**Additional file 2: Table S2.** Sequencing information summary: number of raw reads, enzyme reads, and clean reads and percentage of enzyme reads and clean reads

**Additional file 3: Fig. S1.** Microbial diversity and structure of the renal pelvis urobiome among four groups. (a) Comparison of alpha diversity (Chao1, Shannon index, and Simpson index) between SM and SF. (b) Comparison of beta diversity between SM and SF based on Bray-Curtis distance, Binary Jaccard distance and Euclidean distance. (c) Comparison of alpha diversity (Chao1, Shannon index, and Simpson index) between SM and NSM. (d) Comparison of beta diversity between SM and NSM based on Bray-Curtis distance, Binary Jaccard distance and Euclidean distance. (e) Comparison of alpha diversity (Chao1, Shannon index, and Simpson index) between SF and NSF. (f) Comparison of beta diversity between SF and NSF based on Bray-Curtis distance, Binary Jaccard distance and Euclidean distance

**Additional file 4: Table S3.** List of the 20 species in the optimal marker set

## Data Availability

The datasets used and analyzed in this study are available from the corresponding author on reasonable request.

## Abbreviations

KSD: Kidney stone disease
CaOx: Calcium oxalate
CaP: Calcium phosphate
Urobiome: Urinary microbiome
EQUC: Enhanced quantitative urine culture
2bRAD-M: 2BRAD sequencing for Microbiome
UTIs: Urinary tract infections
2b-Tag-DB: 2BRAD tag database
PCoA: Principal coordinate analysis
LDA: Linear discriminant analysis
LEfSe: Linear discriminant analysis effect size
SD: Standard deviation
ROC: Receiver operating characteristic curve
AUC: Area under the ROC curve
POD: Probability of disease
PERMANOVA: Permutational multivariate analysis of variance
SCFAs: Short-chain fatty acids
