# Supplementary material for: The renal pelvis urobiome in the unilateral kidney stone patients revealed by 2bRAD-M
Source: J Transl Med. 2022 Sep 24;20:431. doi: 10.1186/s12967-022-03639-6 (PMC9509602; doi:10.1186/s12967-022-03639-6)
Supplement: Supplementary file 1 — Additional file 1: Table S1. The sequences of adaptors and primers used in 2bRAD-M (5’-3’) [file 12967_2022_3639_MOESM1_ESM.docx]

**Table S1. The sequences of adaptors and primers used in 2bRAD-M (5’-3’)**

| Name | Adaptor sequence |
| --- | --- |
| Adap-1 sense | ACACTCTTTCCCTACACGACGCTCTTCCGATCTNN |
| Adap-2 sense | GTGACTGGAGTTCAGACGTGTGCTCTTCCGATCTNN |
| Adap antisense | AGATCGGAAGAGC |
|  | Primer sequence |
| Primer1 | ACACTCTTTCCCTACACGACGCT |
| Primer2 | GTGACTGGAGTTCAGACGTGTGCT |
| Index primer | CAAGCAGAAGACGGCATACGAGATXXXXXXGTGACTGGAGTTCAGACGTGT |
