# Supplementary material for: The renal pelvis urobiome in the unilateral kidney stone patients revealed by 2bRAD-M
Source: J Transl Med. 2022 Sep 24;20:431. doi: 10.1186/s12967-022-03639-6 (PMC9509602; doi:10.1186/s12967-022-03639-6)
Supplement: Supplementary file 2 — Additional file 2: Table S2. Sequencing information summary: number of raw reads, enzyme reads, and clean reads and percentage of enzyme reads and clean reads [file 12967_2022_3639_MOESM2_ESM.docx]

**Table S2. Sequencing information summary: number of raw reads, enzyme reads, and clean reads and percentage of enzyme reads and clean reads**

| Sample | Raw reads | Enzyme reads (%) | Clean reads (%) |
| --- | --- | --- | --- |
| C1 | 9755910 | 6562023 (67.26) | 6228922 (63.85) |
| C10 | 10462369 | 7655423 (73.17) | 7299236 (69.77) |
| C11 | 12932806 | 10581337 (81.82) | 10110990 (78.18) |
| C12 | 10365438 | 6239475 (60.19) | 5954307 (57.44) |
| C13 | 10099797 | 7653024 (75.77) | 7247779 (71.76) |
| C14 | 10354113 | 7275461 (70.27) | 6912327 (66.76) |
| C15 | 10377079 | 7349967 (70.83) | 7007188 (67.53) |
| C16 | 10128470 | 7138791 (70.48) | 6802293 (67.16) |
| C17 | 11262742 | 7710854 (68.46) | 7303418 (64.85) |
| C18 | 11249879 | 8273122 (73.54) | 7863991 (69.9) |
| C19 | 10190699 | 6769872 (66.43) | 6445408 (63.25) |
| C2 | 10242047 | 6684995 (65.27) | 6363318 (62.13) |
| C20 | 11891588 | 9041048 (76.03) | 8598898 (72.31) |
| C21 | 11973013 | 8822904 (73.69) | 8365773 (69.87) |
| C22 | 10824968 | 7637495 (70.55) | 7260835 (67.07) |
| C23 | 12424250 | 8989232 (72.35) | 8552307 (68.84) |
| C24 | 11430541 | 8865587 (77.56) | 8445276 (73.88) |
| C25 | 8914259 | 6422422 (72.05) | 6079156 (68.2) |
| C26 | 8953666 | 6544038 (73.09) | 6211126 (69.37) |
| C27 | 9621336 | 6284872 (65.32) | 5971066 (62.06) |
| C28 | 7826565 | 5518754 (70.51) | 5244153 (67) |
| C29 | 9952683 | 8113120 (81.52) | 7688959 (77.26) |
| C3 | 11370363 | 9012216 (79.26) | 8579793 (75.46) |
| C30 | 8588988 | 6088314 (70.89) | 5789826 (67.41) |
| C4 | 12419037 | 10660429 (85.84) | 10158205 (81.8) |
| C5 | 10111212 | 8068632 (79.8) | 7654426 (75.7) |
| C6 | 9068658 | 6710322 (73.99) | 6385378 (70.41) |
| C7 | 10751933 | 7340230 (68.27) | 6985035 (64.97) |
| C8 | 12857160 | 10016116 (77.9) | 9533085 (74.15) |
| C9 | 9973314 | 7325055 (73.45) | 6961825 (69.8) |
| S1 | 9471971 | 7275404 (76.81) | 6888872 (72.73) |
| S10 | 11185672 | 8545140 (76.39) | 8136248 (72.74) |
| S11 | 11754823 | 9510374 (80.91) | 9101933 (77.43) |
| S12 | 11239610 | 8858758 (78.82) | 8421793 (74.93) |
| S13 | 9167453 | 7723049 (84.24) | 7325588 (79.91) |
| S14 | 10452981 | 8364190 (80.02) | 7933893 (75.9) |
| S15 | 8028830 | 5413469 (67.43) | 5149724 (64.14) |
| S16 | 9992020 | 6613061 (66.18) | 6273795 (62.79) |
| S17 | 9813033 | 7098212 (72.33) | 6707578 (68.35) |
| S18 | 9918218 | 7283698 (73.44) | 6901281 (69.58) |
| S19 | 10842132 | 7476952 (68.96) | 7113726 (65.61) |
| S2 | 8421935 | 5516394 (65.5) | 5242200 (62.24) |
| S20 | 11700318 | 8955691 (76.54) | 8482054 (72.49) |
| S21 | 8880226 | 7139284 (80.4) | 6737796 (75.87) |
| S22 | 13029181 | 9443389 (72.48) | 8978011 (68.91) |
| S23 | 9737056 | 6814373 (69.98) | 6484780 (66.6) |
| S24 | 11512469 | 8570507 (74.45) | 8124059 (70.57) |
| S25 | 9191910 | 6398124 (69.61) | 6039819 (65.71) |
| S26 | 9478795 | 6481389 (68.38) | 6149181 (64.87) |
| S27 | 8014469 | 6466161 (80.68) | 6149139 (76.73) |
| S28 | 9627617 | 6931436 (72) | 6576672 (68.31) |
| S29 | 10056883 | 8417250 (83.7) | 7953277 (79.08) |
| S3 | 8290692 | 5847384 (70.53) | 5584175 (67.35) |
| S30 | 8675565 | 6130689 (70.67) | 5820969 (67.1) |
| S4 | 9355369 | 7736767 (82.7) | 7337707 (78.43) |
| S5 | 8409925 | 6540583 (77.77) | 6191195 (73.62) |
| S6 | 10034636 | 7087357 (70.63) | 6736652 (67.13) |
| S7 | 8438152 | 5673448 (67.24) | 5405824 (64.06) |
| S8 | 11279182 | 8925517 (79.13) | 8455374 (74.96) |
| S9 | 10199035 | 7602808 (74.54) | 7210894 (70.7) |
