# Supplementary material for: The renal pelvis urobiome in the unilateral kidney stone patients revealed by 2bRAD-M
Source: J Transl Med. 2022 Sep 24;20:431. doi: 10.1186/s12967-022-03639-6 (PMC9509602; doi:10.1186/s12967-022-03639-6)
Supplement: Supplementary file 4 — Additional file 4: Table S3. List of the 20 species in the optimal marker set [file 12967_2022_3639_MOESM4_ESM.docx]

**Table S3. List of the 20 species in the optimal marker set**

| Acinetobacter_junii |
| --- |
| Pseudomonas_putida |
| Stenotrophomonas_sp_LMG_10879 |
| Acinetobacter_sp_CIP_110321 |
| Cupriavidus_metallidurans |
| Acinetobacter_ursingii |
| Acinetobacter_johnsonii |
| Pseudomonas_fluorescens |
| Sphingomonas_sp_S_NIH_Pt1_0416 |
| Pseudomonas_sp_FW104_15G4B |
| Pseudomonas_sp_LAIL14HWK12_I2 |
| Lactobacillus_iners |
| Corynebacterium_sp_HMSC073D01 |
| Corynebacterium_pyruviciproducens |
| Stenotrophomonas_maltophilia |
| Proteus_mirabilis |
| Acinetobacter_sp_MN12 |
| Prevotella_bivia |
| Brevundimonas_sp_DS20 |
| Sphingomonas_paucimobilis |
